# A Mini Review: Can Graphene Be a Novel Material for Perovskite Solar Cell Applications?

**DOI:** 10.1007/s40820-017-0182-0

**Published:** 2017-12-22

**Authors:** Eng Liang Lim, Chi Chin Yap, Mohammad Hafizuddin Hj Jumali, Mohd Asri Mat Teridi, Chin Hoong Teh

**Affiliations:** 1 School of Applied Physics, Faculty of Science and Technology, Universiti Kebangsaan Malaysia, 43600 Bangi, Selangor Malaysia; 2 Solar Energy Research Institute, Universiti Kebangsaan Malaysia, 43600 Bangi, Selangor Malaysia; 3 ASASI Pintar Program, Pusat Permata Pintar Negara, Universiti Kebangsaan Malaysia, 43600 Bangi, Selangor Malaysia

**Keywords:** Perovskite solar cells, Graphene, Conductive electrode, Carrier transporting material, Stabilizer material, Performance and stability

## Abstract

Perovskite solar cells (PSCs) have raised research interest in scientific community because their power conversion efficiency is comparable to that of traditional commercial solar cells (i.e., amorphous Si, GaAs, and CdTe). Apart from that, PSCs are lightweight, are flexible, and have low production costs. Recently, graphene has been used as a novel material for PSC applications due to its excellent optical, electrical, and mechanical properties. The hydrophobic nature of graphene surface can provide protection against air moisture from the surrounding medium, which can improve the lifetime of devices. Herein, we review recent developments in the use of graphene for PSC applications as a conductive electrode, carrier transporting material, and stabilizer material. By exploring the application of graphene in PSCs, a new class of strategies can be developed to improve the device performance and stability before it can be commercialized in the photovoltaic market in the near future.
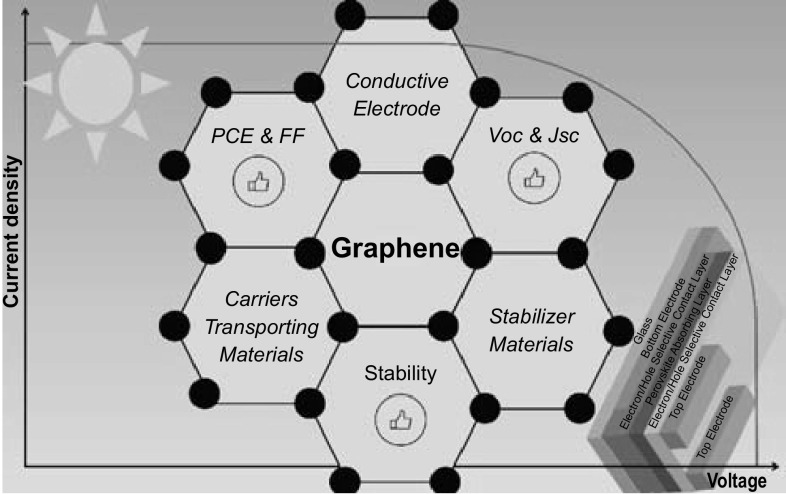

## Highlights

- Introduction of graphene improves photovoltaic properties of perovskite solar cells (PSCs).
- Graphene can be used as a conductive electrode, carrier transporting material, or stabilizer material.
- Graphene enhances the electrical properties and stability of PSCs.

## Introduction

Global climate change [1] and rapidly rising energy demand [2] require society to move toward sustainable and renewable energy resources. Among all sustainable and renewable energy resources, solar energy has potential to fulfill these needs because it is free and clean. Therefore, photovoltaic cells are extremely important for the conversion of solar energy into electricity. According to the latest survey, 90% of the photovoltaic products in the world market are based on first-generation crystalline (monocrystalline and polycrystalline) silicon (Si) wafers with power conversion efficiency (PCE) between 15 and 20% on the module level of 1.6 m^2^ [3]. However, these cells are expensive due to the high cost of the processing and raw material of Si [4]. On the other hand, second-generation solar cells based on amorphous Si, cadmium telluride (CdTe), and copper indium gallium selenide (CIGS) did not repeat the success of the crystalline Si solar cells due to technological problems and the module stability issues [5, 6].

**Fig. 1:**
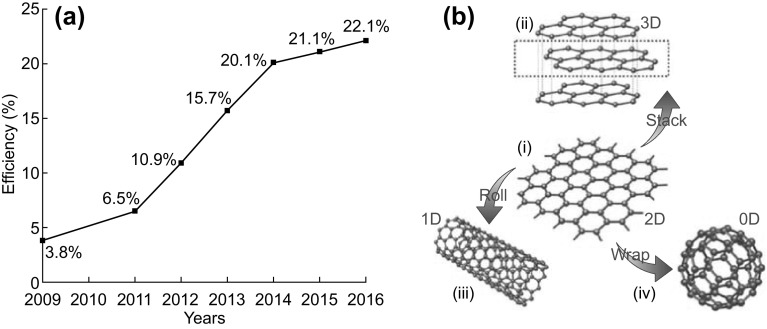
**a** Evolution of PSC efficiencies from 2009 to 2016 [8–14]. **b** Graphene is a 2D hexagonal lattice of carbon atoms (i). It can be stacked into 3D graphite (ii), rolled into 1D nanotubes (iii), and wrapped into 0D buckyballs (iv). Reprinted with permission from Ref. [41] Copyright 2012 American Chemical Society

To overcome these problems, researchers need to explore new materials for next-generation photovoltaics. At present, perovskite solar cells (PSCs) have generated broad interest because of their rapid PCE improvement from 3.8% in 2009 to 22.1% in 2016 [7], as shown in Fig. 1a [8–14]. In addition, PSCs have the merits of low-cost processing, easy fabrication, and compatibility with flexible plastic substrates for large-area production [15, 16]. It should be noted that the certified efficiency of 16.0 ± 0.4% has been achieved for the minimodule PSC with the aperture area of 16.29 cm^2^ [17]. Thus, PSCs have been considered as promising energy conversion candidates for carbon-free energy production in the next few years.

To date, several strategies such as electrode modification [18–21], metal-doping cathode buffer layer [22–25], surface modified perovskite layer [26–28], plasmonic nanoparticles [29–32], and introducing graphene-based materials within the device layers [33–37] have been carried out to boost the performance and stability of PSCs. In particular, integration of graphene into PSCs has attracted attention because graphene provides promising device designs with its potential low cost of production, high chemical stability, and appropriate energy level [38]. It should be noted that the efficiency of PSCs of over 18% has been achieved by using graphene-based materials (the highest reported efficiency of graphene PSCs to date) [39], indicating that graphene is a promising candidate for the development of PSCs. Graphene was discovered in 2004 by Russian-born scientists Andre Geim and Konstantin Novoselov utilizing, in the widely accepted terminology, the “Scotch tape method” [40].

It is well known that graphene is a name given to a two-dimensional (2D) sheet of *sp* ^2^ hybridized carbon atoms tightly packed into a 2D honeycomb crystal lattice. It can be stacked to form three-dimensional (3D) graphite, rolled to form one-dimensional (1D) nanotubes, and wrapped to form zero-dimensional (0D) fullerenes. Figure 1b illustrates graphene and its derivatives [41]. Its outstanding properties such as high thermal conductivity (~ 5 × 10^3^ W m^−1^ K^−1^) at room temperature [42], high charge carrier mobility (2 × 10^5^ cm^2^ V^−1^ S^−1^) [43], high optical transparency (it absorbs 2.3% of the incident light in the range from infrared to violet) [44], large surface-to-mass ratio (2630 m^2^ g^−1^) [45], superior mechanical properties (i.e., Young’s modulus of ~ 1 TPa) [46], and high flexibility have given it the potential to be used in various applications such as sensors [47], catalysts [48], optical modulators [49], surface-enhanced Raman spectroscopy (SERS) platforms [50], and optoelectronic devices (light-emitting diodes, solar cells, displays, touch screens) [51, 52].

Here, we present the successful application of graphene for high-performance and stable PSCs. Firstly, we introduce the crystal structure and tolerance factor of organic–inorganic perovskite materials. Secondly, we briefly introduce the working principle of PSCs. Thirdly, we highlight the progress of applying graphene for high-performance and stable PSCs. Finally, we provide the outlook and conclusion on the application of graphene for PSCs.

## Crystal Structure and Tolerance Factor of Organic–Inorganic Perovskite Materials

**Fig. 2:**
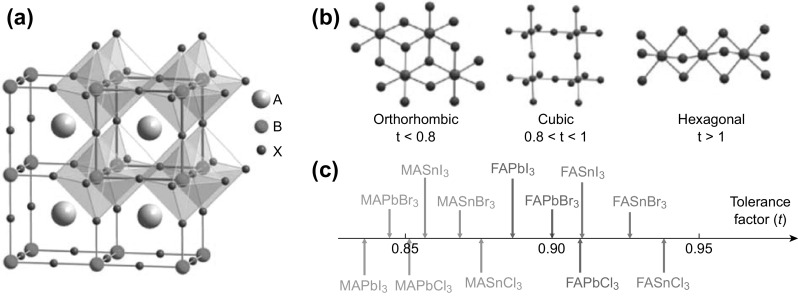
**a** Basic ABX_3_ perovskite crystal structure, reprinted with permission from [55] © 2008 American Physical Society. **b** Relationship between the tolerance factor, t, and the crystal structure of perovskite materials, reprinted with permission from [56] © 2016 American Chemical Society. **c** Tolerance factor of the different perovskite materials for perovskite solar cells applications, reprinted with permission from Ref. [58] Copyright 2015 The Royal Society of Chemistry

The term “perovskite” is used when referring to a large compound group that has the same crystal structure as mineral perovskite CaTiO_3_ [53]. For perovskite materials in solar energy applications, the basic building component is an ABX_3_ crystal structure, where A represents an organic and/or inorganic cation such as methylammonium (CH_3_NH_3_^+^ or MA^+^), formamidinium (NH(CH_3_)_2_^+^ or FA^+^), or cesium (Cs^+^), B represents a divalent metal cation (Pb^2+^ or Sn^2+^), and X represents a halide anion (Cl^−^, Br^−^, or I^−^) [13, 54]. The structures of the ABX_3_ type are shown in Fig. 2a [55]. For a stable and formable ABX_3_ perovskite structure, the tolerance factor, *t* = (*R*_A_ + *R*_X_)/{√2(*R*_B_ + *R*_X_)} of perovskite materials should be close to 1 (corresponding to a perfectly packed perovskite structure), where *R*_A_, *R*_B_, and *R*_X_ are the effective ionic radii for A, B, and X ions, respectively.

It has been reported that organic–inorganic hybrid halide perovskite materials tend to form an orthorhombic structure if *t* < 0.8, cubic structure if 0.8 < *t* < 1.0, and hexagonal structure if *t* > 1 [56], as shown in Fig. 2b. Based on the tolerance factor equation, the effective radii of Pb^2+^, Sn^2+^, Cl^−^, Br^−^, and I^−^ are 1.19, 1.18, 1.84, 1.96, and 2.20 Å [57], respectively. It is suggested that the organic or inorganic cation A with radius between 1.60 and 2.50 Å can form stable lead and/or tin halide perovskite structures at 0.8 < *t* < 1.0 (see Table 1). Figure 2c shows the tolerance factor of the series of perovskite materials [58] that have been used for an absorbing layer in PSC applications. Other than the tolerance factor, the ionization energy of the organic–inorganic cation [59], dimensional phases of perovskite materials [60], chemical stability, valence band [58], etc., are also important for the formability and stability of perovskite structures.

**Table 1:** Estimation of A cation radii based on lead and/or tin trihalides perovskite materials, ABX_3_

*R*_B_ (Å)	*R*_X_ (Å)	*R*_A_^#^ (Å) at *t* = 0.8	*R*_A_^#^ (Å) at *t* = 1.0
Pb^2+^ (1.19)	Cl^−^ (1.84)	1.59	2.44
	Br^−^ (1.96)	1.60	2.49
	I^−^ (2.20)	1.63	2.59
Sn^2+^ (1.18)	Cl^−^ (1.84)	1.58	2.43
	Br^−^ (1.96)	1.59	2.48
	I^−^ (2.20)	1.62	2.58

^#^ *R*_A_ (Å) = *t*({√2(*R*_B_ + *R*_X_)}) − *R*_x_ (Å)

## Working Principle of PSCs

In general, the perovskite layer is composed of organic materials such as organic cations (i.e., methylammonium, ethylammonium, formamidinium) and inorganic materials such as metal cations (i.e., Pb^2+^, Sn^2+^, and Ge^2+^) and halide anions (i.e., I^−^, Cl^−^, and Br^−^). As a result, the working principle of PSCs has raised a number of questions because the optical absorption of the perovskite layer cannot be distinguished. It should be noted that at the beginning of its discovery, PSCs were TiO_2_-sensitized solar cells, using MAPbI_3_ and MAPbBr_3_ as sensitizers [8]. The free electrons excited after photon absorption by the sensitizer would be injected into the conduction band of the wide band gap TiO_2_ inorganic semiconductor, followed by electron extraction to the transparent conductive oxide (TCO). The electrons would flow through the external circuit to the platinum (Pt) anode and then into the iodide electrolyte. The electrolyte would regenerate and transport the electrons back to the dye molecules. Therefore, it has been proposed that dye-sensitized solar cells (DSSCs) can be used as a model to explain the working mechanism of PSCs. Because of the dissolution issues of halides in a liquid electrolyte that could influence the stability of PSCs [9], the liquid electrolyte was replaced by a solid-state hole transporting material (i.e., Spiro-OMeTAD) [10].

**Fig. 3:**
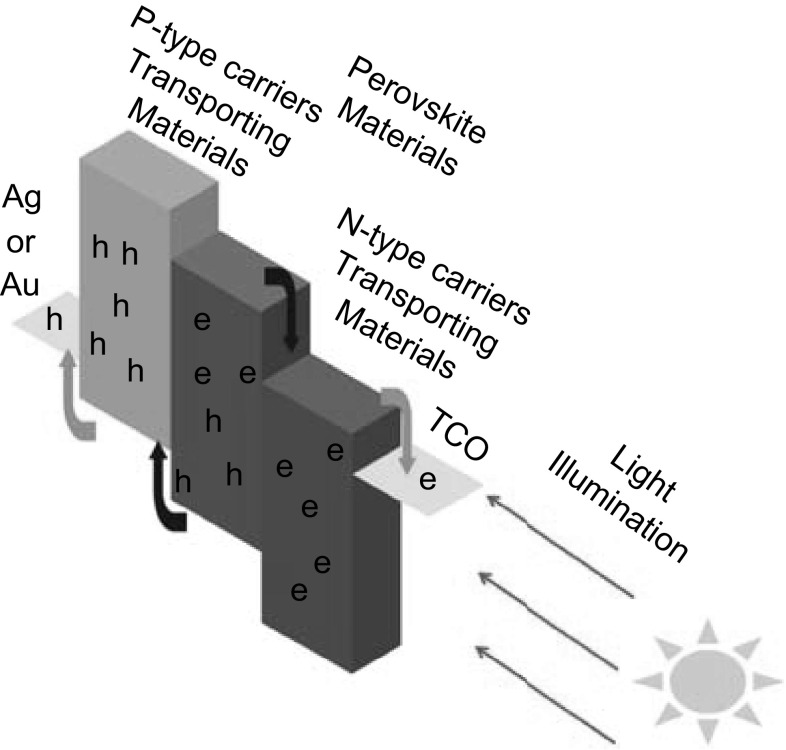
General working mechanism of PSCs. Free charge carriers formed in the perovskite layer drift to the carrier transporting material (black arrow line), followed by charge extraction to the electrode (blue arrow line). (Color figure online)

In the case of PSCs, the perovskite material itself is an intrinsic (neither p-type nor n-type) semiconductor. Owing to the low binding energy of perovskite materials (2–55 meV [61, 62]), the free charge carriers (free electrons and free holes) formed inside the perovskite layer after photon absorption [61] can be quickly injected into electron/hole transporting materials with very slow charge carrier recombination and result in large values of the diffusion length [63]. Finally, the electrons/holes are extracted to the cathode/anode. Figure 3 illustrates the basic working mechanism of PSCs.

## Discussion

### Graphene as Conductive Electrode

**Fig. 4:**
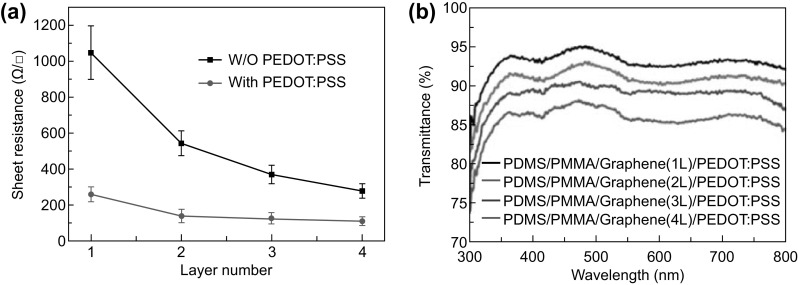
**a** Sheet resistance and **b** transmittance spectra of CVD-produced graphene films (one to four layers) before and after being doped with PEDOT:PSS. Reprinted with permission from Ref. [65] Copyright 2015 John Wiley & Sons, Inc

Recently, graphene has been successfully used as a conductive electrode for PSC applications [35, 64–67]. This was first reported by Yan et al. [65] using the lamination method for the chemical vapor deposition (CVD) produced graphene. Due to the high sheet resistance (~ 1050 ± 150 Ω sq^−1^) of single-layer CVD-produced graphene films, a thin layer (~ 20 nm) of poly-(3,4-ethylenedioxythiophene):poly(styrenesulfonate) (PEDOT:PSSS) solution doped with fluorosurfactant Zonyl-FS300 together with D-sorbitol was spin-coated on top of the graphene surface to (1) reduce the graphene sheet resistance (2) act as an adhesion layer during the lamination process, and (3) induce more hole doping to the graphene electrode, since the Fermi level of PEDOT:PSS is higher than the graphene Dirac point. After optimizing the processing condition, the device performance with double-layer graphene films could achieve up to 12.37% efficiency, which is relatively high compared to that of the reported semitransparent TCO-free PSCs [68, 69]. The superior performance and high *J*_sc_ of the champion device originated from the low sheet resistance (Fig. 4a) and higher conductivity of graphene electrode after it was coated with PEDOT:PSS, as well as the high transmittance of the thin films in the visible spectral region (*T* > 90%, see Fig. 4b) [65].

**Fig. 5:**
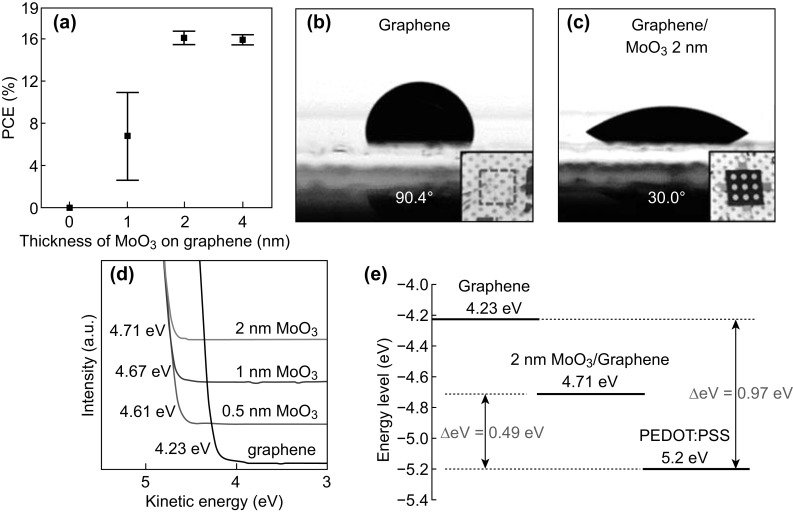
**a** Average device performance with various thicknesses of MoO_3_ HTM on graphene **b** Contact angle measurement using PEDOT:PSS on pristine graphene surface and **c** 2 nm of MoO_3_/graphene surface. The insets in **b** and **c** are the optical images of PEDOT:PSS/MAPbI_3_ films coated on the corresponding graphene-coated glass substrate. The MoO_3_ layers were deposited in a square shape at the center of the substrates before spin-coating of PEDOT:PSS/MAPbI_3_ thin films. **d** Work function of the graphene surface before and after being coated with MoO_3_ HTM, which has been extracted from the UPS spectra. Reprinted with permission from Ref. [35] Copyright 2016 John Wiley & Sons, Inc. **e** Energy level alignment of PEDOT:PSS, graphene, and 2 nm MoO_3_/graphene

On the other hand, Choi et al. [35] investigated a single-layer graphene-coated glass substrate acting as a transparent bottom anode. The authors discovered that the performance of the device with a structure of graphene-coated glass substrate/PEDOT:PSS/MAPBI_3_/C_60_/BCP/LiF/Al could not be evaluated (see Fig. 5a) because neither PESOT:PSS nor perovskite solutions could wet the hydrophobic graphene surface to form a uniform thin film (see Fig. 5b). Through interfacial engineering by incorporating 2 nm of molybdenum trioxide (MoO_3_) hole transporting material (HTM) on top of the graphene electrode surface, the device performance could be improved to achieve efficiencies of up to 17.1%. The dramatic increase in the device performance was attributed to (1) the use of MoO_3_ HTM, which provided hydrophilicity to the graphene surface (see Fig. 5c) and (2) the formation of desirable energy level alignment between the MoO_3_/graphene electrode and PEDOT:PSS (see Fig. 5d, e).

**Fig. 6:**
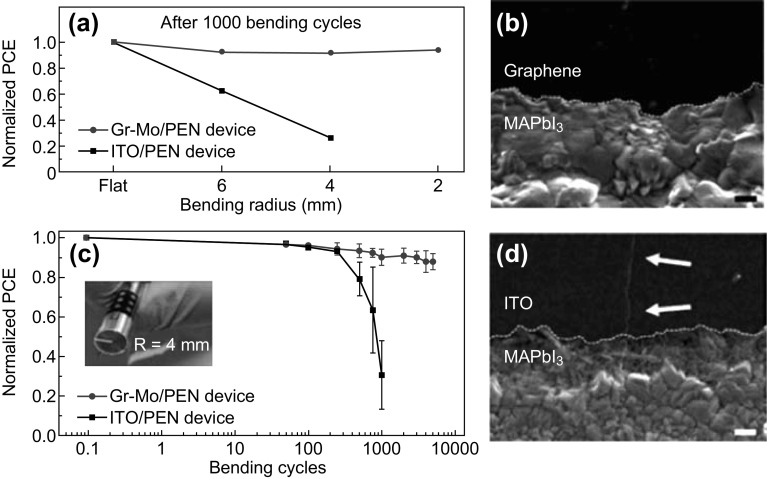
Normalized PCE of graphene-MoO_3_/PEN and ITO/PEN devices measured **a** after 1000 bending cycles at radii of flat, 2, 4, and 6 mm and **c** as a function of bending cycles at a fixed bending radius of 4 mm. Cross-sectional SEM images of MAPBI_3_ perovskite films coated on **b** PEN/ITO/PEDOT:PSS and **d** PEN/Graphene-MoO_3_/PEDOT:PSS after 1000 bending cycles at the bending radius of 4 mm. The scale bar is 200 nm. Reprinted with permission from Ref. [67] Copyright 2017 The Royal Society of Chemistry

Later, Choi et al. [67] used the same structure on a flexible polyethylene naphthalate (PEN) substrate (PEN/graphene**/**MoO_3_/PEDOT:PSS/MAPbI_3_/C_60_/BCP/LiF/Al) and evaluated its operational stability against repeated bending. Under strain-free conditions, the device with a graphene conductive electrode exhibited a PCE of 16.8%, which was kept within ~ 90% of the initial value after it had been bent 1000 times at *R* = 6, 4, and 2 mm (see Fig. 6a). This can be attributed to the use of the graphene-based conductive electrode; as a result, no signs of damage were observed on the graphene or perovskite thin film surfaces under bending conditions (see Fig. 6b). In contrast, indium-doped tin oxide (ITO)-based flexible PSCs [70, 71] showed a rapid decrease in PCE after they had been bent more than 250 times at *R* = 4 mm (see Fig. 6c); this was mainly attributed to the emergence of cracks on the brittle ITO surface, which later diffused into the perovskite thin films (see Fig. 6d). In comparison with the PEN/ITO substrate (sheet resistance *R*_sheet_ = 13.3 ± 1.3 Ω sq^−1^), the graphene-MoO_3_/PEN substrate showed a higher sheet resistance of 552.0 ± 24.2 Ω sq^−1^, which could reduce the charge collection efficiency of the device, leading to a high series resistance, low shunt resistance, and low the fill factor (FF). However, both devices showed similar *J*_sc_ values; this can be attributed to the use of the graphene-based substrate exhibiting higher transmission (~ 97% transmittance) compared to the PEN/ITO-based substrate (~ 89% transmittance) over the visible wavelength region.

On the other hand, Liu et al. [72] demonstrated a flexible PSC with a poly(3-hexylthiophene-2,5-diyl) P3HT HTM-coated graphene transparent electrode on top of a 20-μm-thick polyethylene terephthalate (PET) substrate. P3HT was used as HTM because its highest occupied molecular orbital (HOMO) level of ≈ − 5.2 eV is much closer to the valence band of MAPbI_3_ (≈ − 5.4 eV) than that of conventional HTMs such as PEDOT:PSS (≈ − 5.0 eV), which can facilitate hole transfer to the anode. Furthermore, it can also enhance the device stability owing to the hydrophobic nature of P3HT that does not absorb moisture in air [73, 74]. After optimizing the processing conditions by introducing ~ 2-μm cross-linkable olefin-type polymer (ZEOCOAT™) as an interlayer prior to transferring the graphene layer, the device exhibited higher performance (11.5%) compared to a control device (10.4%), suggesting that the ZEOCOAT™ interlayer reduced the surface roughness of the PET substrate. In addition, it was found under strain conditions that flexible devices can operate at different bending radii (*R* = 0.670, 0.365, 0.175, and 0.130 cm) with 14% degradation in device performance at the bending radius of 0.175 cm after 500 cycles [66]. Later, Heo et al. [64] demonstrated that gold chloride (AuCl_3_)-doped graphene anode surface could extend the diffusion length of holes to the graphene electrode from ~ 210 nm (control device) to ~ 370 nm (champion device), where the champion device performance was enhanced by 42.1% compared to the control device.

### Graphene as Carrier Transporting Material

**Fig. 7:**
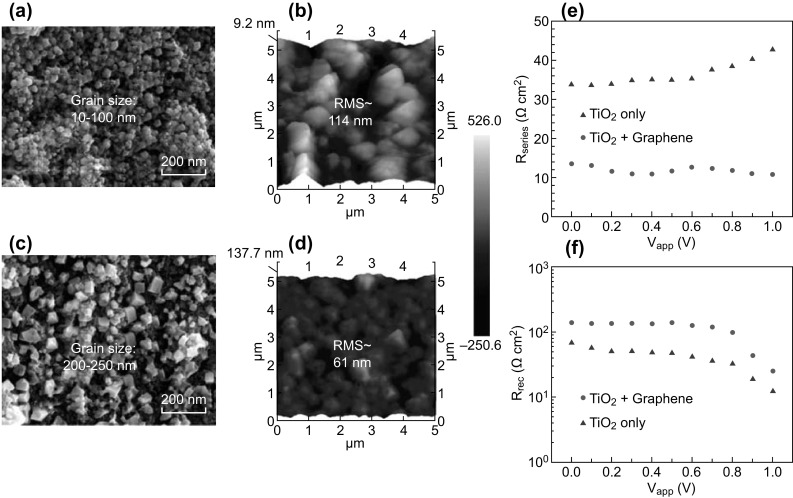
SEM and AFM images of perovskite thin films prepared on ZnO films (**a**, **b**) and 0.75 wt% graphene/ZnO nanocomposite (**c**, **d**) coated glass substrates. Reprinted with permission from Ref. [75] Copyright 2017 The Royal Society of Chemistry. Series resistance **e** and recombination resistance **f** obtained from impedance spectroscopy analysis of two samples with different concentrations of graphene in TiO_2_ ESCL. Reprinted with permission from Ref. [77] Copyright 2014 American Chemical Society

Graphene has also been used as a carrier transporting material for PSC applications. For example, Chandrasekhar et al. [75] demonstrated that the PCE of ZnO-based PSCs could be enhanced by 47.5% from 7.01 ± 0.66% up to 10.34 ± 0.18% after graphene has been doped into the ZnO electron selective contact layer (ESCL). According to the authors, the increase in the device performance up to 48.0% can be attributed to the formation of a superior perovskite thin film (larger grains size with low surface roughness, see Fig. 7a–d) on the graphene network in the ZnO nanocrystal, which enhanced the charge carrier mobility by reducing the charge carrier recombination at the defect and trap states within the perovskite layer [76]. On the other hand, Snaith et al. [77] employed graphene nanoflakes/TiO_2_ nanocomposite as the ESCL and demonstrated the enhancement of the device performance (PCE = 15.6%) by 56.0% compared to the device with pure TiO_2_ thin films (PCE = 10.0%).

To evaluate the role of graphene addition into TiO_2_ layers, the authors performed impedance spectroscopy characterization under working cell conditions and observed that the addition of graphene into TiO_2_ thin films could effectively reduce the series resistance of the device (see Fig. 7e) and charge carrier recombination rate (see Fig. 7f). In addition to binary oxides (ZnO and TiO_2_), Wang et al. [78] demonstrated a graphene-doped ternary oxide-based PSC with a structure of glass/fluorine-doped tin oxide (FTO)/TiO_2_/graphene-SrTiO_3_/MAPbI_3_/Spiro-OMeTAD/Ag. The authors showed that graphene could enhance the electron transfer rate. As a result, the optimized device exhibited a PCE of 10.49%, which was enhanced by 53.1% compared to that of a control device with a PCE of 6.85%.

To date, the highest efficiency of graphene-based PSCs has been 18.19%, which was reported by Agresti et al. [34], with an architecture of FTO-coated glass substrate/compact TiO_2_/graphene-doped mesoporous TiO_2_/MAPbI_3_/graphene oxide/Spiro-OMeTad/gold. The high efficiency of the device was mainly attributed to the improved charge carrier injection/extraction and the device stability [39].

### Graphene as Stabilizer Material

**Fig. 8:**
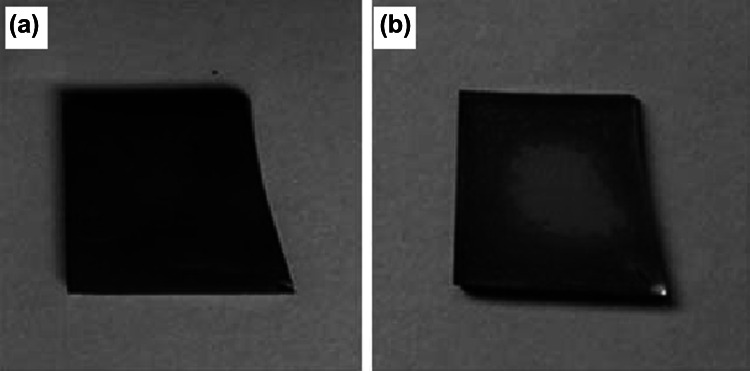
Photograph images of perovskite thin films **a** before and **b** after decaying. Reprinted with permission from Ref. [79] Copyright 2016 Nature Publishing Group

In addition, it is well known that perovskite thin films can be easily hydrolyzed and decomposed from dark brown into yellowish thin films under humid air environment (see Fig. 8a, b). The degradation process and decay mechanism can be described with the following equations [79]:

1 $${\text{CH}}_{3} {\text{NH}}_{3} {\text{PBI}}_{3} + {\text{H}}_{2} {\text{O}} \leftrightarrow {\text{CH}}_{3} {\text{NH}}_{3} {\text{PBI}}_{3} \cdot {\text{H}}_{2} {\text{O}}$$

2 $${\text{CH}}_{3} {\text{NH}}_{3} {\text{PBI}}_{3} \cdot {\text{H}}_{2} {\text{O}} \leftrightarrow {\text{PBI}}_{2} + {\text{CH}}_{3} {\text{NH}}_{3} {\text{I}} + {\text{H}}_{2} {\text{O}}$$

In order to solve the problem, deposition of an additional layer on top of the perovskite layer was proposed, using either a graphene layer [80] or doped with ESCL [81] /holes selective contact layer (HSCL) [82]. This is because the hydrophobic nature of graphene results in the weak affinity to moisture, and therefore, the layer can prevent the perovskite thin films from reacting with moisture in air. As a result, the diffusion of halide ions from the perovskite layer to the top electrode (Ag or Au electrode) can be inhibited [81]. The lattice parameter of 2D graphene is 0.246 nm [83], which is much smaller than the diameters of all halide anions (see Table 1). Therefore, graphene can also block the diffusion of Ag or Au atoms from the metal electrode to the perovskite thin films through the hole transporting layer [84, 85] during the thermal treatment process at ambient environment. As a result, the accordingly fabricated devices showed very good thermal and optical stabilities.

For example, Hu et al. [80] reported that CVD-produced graphene on top of perovskite/Spiro-OMeTAD thin films could retain > 94% of its original efficiency after it has been kept in 45% humidity air for 96 h or after thermal annealing at 80 °C for 12 h, even though graphene itself might reduce the hole extraction to the anode because of its lower work function of ~ 4.2 eV [35] compared to that of Spiro-OMeTAD (~ 5.2 eV) [65]. The *J*_sc_, *V*_oc_, FF, and PCE of the devices with (without) graphene on top of the perovskite/Spiro-OMeTAD thin films are 21.1 (21.6) mA cm^−2^, 1.09 (1.07) V, 0.682 (0.718), and 15.7 (16.6) %, respectively. Bi et al. [81] reported that graphene-doped PCBM-based PSCs could achieve stable efficiencies of over 15% during a thermal aging test at 85 °C for 500 h or light soaking under AM 1.5 G illumination for 1000 h. Cao et al. [82] reported that graphene-doped perthiolated tri-sulfur-annulated hexa-peri-hexabenzocoronene, TSHBC, could retain 90% of its original efficiency after the device has been stored in air with a relative humidity of ~ 45% for 10 days.

## Outlook and Conclusions

In conclusion, graphene has been used as a conductive electrode, carrier transporting material, and stabilizer material for PSC applications (Table 2). So far, CVD has been a common method to produce graphene thin films. This is because the CVD method can produce large-area graphene films and can transfer graphene onto a target substrate either by a roll-to-roll process or spin-coating method to form a conductive electrode even though it involves high-vacuum processing.

**Table 2:** Recent development of graphene-based PSC device performance

Structure	*J*_sc_ (mA cm^−2^)	*V*_oc_ (V)	FF (%)	*η* (%)	Refs.
*Conductive electrode*					
Glass/graphene/MoO_3_/PEDOT:PSS/MAPbI_3_/C_60_/BCP/LiF/Al	21.9^a^	1.03^a^	0.72^a^	16.1^a^	[35]
				17.1^b^	
Glass/AuCl_3_-doped graphene/PEDOT:PSS/MAPbI_3_/PCBM/Al	21.0^b^	1.09^b^	0.783^b^	17.9^b^	[64]
Glass/FTO/TiO_2_/MAPbI_3−x_Cl_x_/Spiro-OMeTAD/PEDOT:PSS/graphene/PMMA/PDMS	19.17^b^	0.960^b^	0.6722^b^	12.37^b^	[65]
				12.02^a^	
PET/ZEOCOAT™/graphene/P3HT/MAPbI_3_/PC_71_BM/Ag	18.58^b^	1.04^b^	59.4^b^	11.48^b^	[66]
				11.27^a^	
PEN/graphene/MoO_3_/PEDOT:PSS/MAPbI_3_/C_60_/BCP/LiF/Al	21.0^a^	0.99^a^	0.72^a^	15.0^a^	[67]
	21.7^b^	1.00^b^	0.78^b^	16.8^b^	
*Carriers transporting material (CTM)*					
Glass/FTO/compact TiO_2_/graphene-doped mesoporous TiO_2_/MAPbI_3_/graphene oxide/Spiro-OMeTAD/Au	22.48^b^	1.08^b^	0.7512^b^	18.19^b^	[39]
				15.42^a^	
Glass/FTO/ZnO/graphene-doped ZnO/MAPbI_3_/Spiro-OMeTAD/Ag	19.97^a^	0.926^a^	0.5631^a^	10.34^a^	[75]
Glass/FTO/graphene-doped TiO_2_/Al_2_O_3_/MAPBI_3−x_Cl_x_/Spiro-OMeTAD/Au	21.9^b^	1.04^b^	0.73^b^	15.6^b^	[77]
Glass/FTO/compact TiO_2_/mesoporous graphene/SrTiO_3_/MAPbI_3_/Spiro-OMeTAD/Ag	18.10^b^	1.00^b^	0.58^b^	10.49^b^	[78]
*Stabilizer materials (SM)*					
Glass/FTO/TiO_2_/MAPbI_3_/Spiro-OmeTAD/graphene/Au	21.1^b^	1.09^b^	0.682^b^	15.7^b^	[80]
Glass/FTO/NiMgLiO/MAPbI_3_/graphene-doped PCBM/CQD/Ag	19.7^a^	1.07^a^	0.751^a^	15.8^a^	[81]
	20.6^b^	1.08^b^	0.766^b^	17.0^b^	
Glass/FTO/compact TiO_2_/mesoporous TiO_2_/MAPbI_3_/graphene-coated TSHBC-R/Au	21.91^b^	0.97^b^	0.66^b^	14.02^b^	[82]

^a^Average value

^b^Best value

In comparison with conventional TCO-based electrodes such as FTO and indium-doped tin oxide (ITO), graphene electrodes show high flexibility and good physical, chemical, and thermal stability. Therefore, the PCE and lifetime of the devices based on graphene electrodes are much better than those of the devices based on TCO electrodes. Apart from that, graphene can enhance the electrical properties of the devices and crystallinity of the perovskite films if graphene is n-doped into metal oxide layers to act as a carrier transporting material.

To further improve the stability of PSCs, it is suggested to add a layer of graphene on top of the perovskite/hole transporting layer thin film. We believe that graphene will play an important role in PSCs in the near future. Therefore, development of novel layered graphene in different layer designs of PSCs with a controlled mechanism will be needed.

We propose to utilize oxidized or reduced graphene, in addition to pristine graphene, for PSC applications. We believe that, by optimizing the deposition process in terms of time, temperature, solvent, precursor selection, etc., new designs of PSCs with good performance and stability can be developed. Once graphene-based materials have proved to be useful in photovoltaic technologies, low-cost PSCs can be realized and commercialized in the market. Further, other optoelectronic devices will be designed follow this trend, initiating the era of low-cost optoelectronic devices.
